# MP2RAGEME: T_1_, T_2_^*^, and QSM mapping in one sequence at 7 tesla

**DOI:** 10.1002/hbm.24490

**Published:** 2018-12-13

**Authors:** Matthan W. A. Caan, Pierre‐Louis Bazin, José P. Marques, Gilles de Hollander, Serge O. Dumoulin, Wietske van der Zwaag

**Affiliations:** ^1^ Spinoza Centre for Neuroimaging Amsterdam The Netherlands; ^2^ Amsterdam UMC University of Amsterdam, Biomedical Engineering and Physics Amsterdam The Netherlands; ^3^ Social Brain Laboratory Netherlands Institute for Neuroscience Amsterdam The Netherlands; ^4^ Max Planck Institute for Human Cognitive and Brain Sciences Leipzig Germany; ^5^ Donders Institute for Brain, Cognition and Behaviour Nijmegen The Netherlands; ^6^ Experimental Psychology, Helmholtz Institute Utrecht University Utrecht The Netherlands; ^7^ Experimental and Applied Psychology VU University Amsterdam The Netherlands

**Keywords:** QSM, quantitative imaging, T_1_ mapping, T_2_^*^ mapping

## Abstract

Quantitative magnetic resonance imaging generates images of meaningful physical or chemical variables measured in physical units that allow quantitative comparisons between tissue regions and among subjects scanned at the same or different sites. Here, we show that we can acquire quantitative T_1_, T_2_^*^, and quantitative susceptibility mapping (QSM) information in a single acquisition, using a multi‐echo (ME) extension of the second gradient‐echo image of the MP2RAGE sequence. This combination is called MP2RAGE ME, or MP2RAGEME. The simultaneous acquisition results in large time savings, perfectly coregistered data, and minimal image quality differences compared to separately acquired data. Following a correction for residual transmit B_1_^+^‐sensitivity, quantitative T_1_, T_2_^*^, and QSM values were in excellent agreement with those obtained from separately acquired, also B_1_^+^‐corrected, MP2RAGE data and ME gradient echo data. The quantitative values from reference regions of interests were also in very good correspondence with literature values. From the MP2RAGEME data, we further derived a multiparametric cortical parcellation, as well as a combined arterial and venous map. In sum, our MP2RAGEME sequence has the benefit in large time savings, perfectly coregistered data and minor image quality differences.

## 1 INTRODUCTION

Quantitative magnetic resonance imaging (MRI) is becoming a popular tool in neuroimaging, in a large part due to the increasing availability of 7 T MR scanners, where the increased signal‐to‐noise ratio (SNR) accommodates more complex and generally longer acquisitions (van der Zwaag, Schäfer, Marques, Turner, & Trampel, 2016). With quantitative MRI, we depart from volumetric representations of the underlying tissues, and obtain a directly comparable measure of more tissue‐specific MRI quantities that may not be captured by studying volume alone (Draganski et al., 2011). With such measures, we can study changes due to aging, disease, or learning‐induced plasticity with increased specificity (Keuken et al., 2017; Tardif et al., 2016; Vargas et al., 2018; Yeatman, Wandell, & Mezer, 2014). Moreover, the measures are more directly reproducible across scanners and sites, even for different sequences (Stikov et al., 2015; Weiskopf et al., 2013).

The main compounds that can be quantitatively measured in the brain are myelin and iron. In white matter (WM), the longitudinal relaxation rate R_1_ = 1/T_1_ has been shown to be linearly related to myelin concentration (Stüber et al., 2014) and axon diameter (Harkins et al., 2016). In gray matter, both R_2_^*^ and quantitative susceptibility mapping (QSM) are linearly related to iron concentration (Deistung et al., 2013a). Cortical gray matter shows a spatial distribution of both myelin and iron, apparent in T_1_‐ and T_2_^*^‐maps (Waehnert et al., 2016).

While T_1_ contrast is used widely to define the gray‐WM border along the cortical surface (Bazin et al., 2014; Fischl et al., 2002), T_2_^*^ and derived contrasts are widely used to segment deep gray matter structures (Keuken, Isaacs, Trampel, van der Zwaag, & Forstmann, 2018).

T_1_ relaxometry can be done with multiple 3D FLASH (fast low angle shot) acquisitions (Frahm, Haase, & Matthaei, 1986) with variable excitation flip angles (Helms, Dathe, & Dechent, 2008). An additional B_1_‐map may be obtained to correct for transmit field (B_1_^+^)‐inhomogeneities, such as by means of a dual refocusing echo acquisition mode in the DREAM‐sequence (Nehrke & Börnert, 2012). Other work performed a segmentation‐based correction, alleviating the need of a separate B_1_‐map (Weiskopf et al., 2011). Acquiring multiple multi‐echo gradient echo (ME‐GRE) readouts enabled quantitative multiparameter mapping of R_1_ and R_2_^*^ (Weiskopf et al., 2013). These parameters could be robustly estimated over multiple sites. In order to reduce the sensitivity to B_1_^+^‐inhomogeneities and alleviate the need to coregister separately acquired volumes, the MP2RAGE‐sequence was proposed. In this inversion‐recovery sequence, two GRE‐readouts follow after optimized inversion times. This allows for high‐resolution imaging at high field (Marques et al., 2010; Marques & Gruetter, 2013). The resulting image is free of T_2_^*^, *M*_0_ (net magnetization or proton density) and B_1_^−^ effects, but a small and protocol‐dependent amount of B_1_^+^ contrast remains. This can be removed by acquiring a B_1_^+^‐map in addition to T_1_‐weighted data (Marques & Gruetter, 2013) and use it in the parameter estimation step. Although the MP2RAGE sequence is robust and widely used at 7 T, the acquisition is relatively inefficient because of the long TR required for the magnetization to return to equilibrium. Longer readouts, containing more k‐space lines, would lead to shorter scan times, but also result in more T_1_‐relaxation during the readout and, hence, incur more T_1_‐induced blurring in the images, leading to a poorer PSF. Therefore, most acquisitions opt for relatively short readouts of typically one k‐space plane, limiting blurring and accepting the acquisition dead time. Optimally exploiting the dead time, as we will propose in this article, will result in a time‐efficient sequence.

Faster imaging at high field was achieved by using a multislice echo planar imaging (EPI) readout (Polders, Leemans, Luijten, & Hoogduin, 2012; van der Zwaag et al., 2018; Wright et al., 2008), but EPI imaging comes at the cost of spatial distortions due to the lower readout bandwidth. Where the MP2RAGE sequence is limited to two readouts, the MPnRAGE performs many more radial readouts with view sharing, sampling the relaxation curve over a wider range (Kecskemeti et al., 2015).

T_2_^*^ relaxometry is commonly performed using a FLASH sequence with a readout comprising of ME. QSM is performed by a dipole deconvolution of the magnetic field, obtained from the phase data of this sequence (Wang & Liu, 2015).

Recent work showed that combined T_1_, T_2_^*^, and QSM mapping is feasible by extending the MP2RAGE sequence to have ME at both readouts (Metere, Kober, Möller, & Schäfer, 2017). This makes efficient use of the dead time in the MP2RAGE sequence, although the ME on the first image following the inversion necessarily leads to a longer readout block and, subsequently, to a nonoptimal inversion time for T_1_‐sensitivity as well as a too‐short TE for optimal T_2_^*^ contrast (TEs should at least match the expected T_2_^*^ values of tissues).

T_1_, T_2_^*^, and QSM are thus widely used contrasts at 7 T and mapping these properties quantitatively is more and more sought after. While indeed many approaches can be used to measure these in reasonable times, challenges arise in terms of inhomogeneities or bias in the estimated quantities and precision of their alignment. Simultaneous acquisition avoids the need for coregistration and subsequent resampling of separately acquired scans and allows time savings while compromises to both maps are minimal.

Here, we present an extension of the MP2RAGE sequence in which ME are acquired on the second inversion. This allows measurement of T_1_, T_2_^*^, and QSM simultaneously and efficiently, with optimized inversion and longer echo times, at high resolution in maximally 17 min, reducing the sequence dead time to 6%, and show that the measured quantities agree well with expected measures after correction of B_1_^+^‐inhomogeneities.

## 2 METHODS

**Figure 1:**
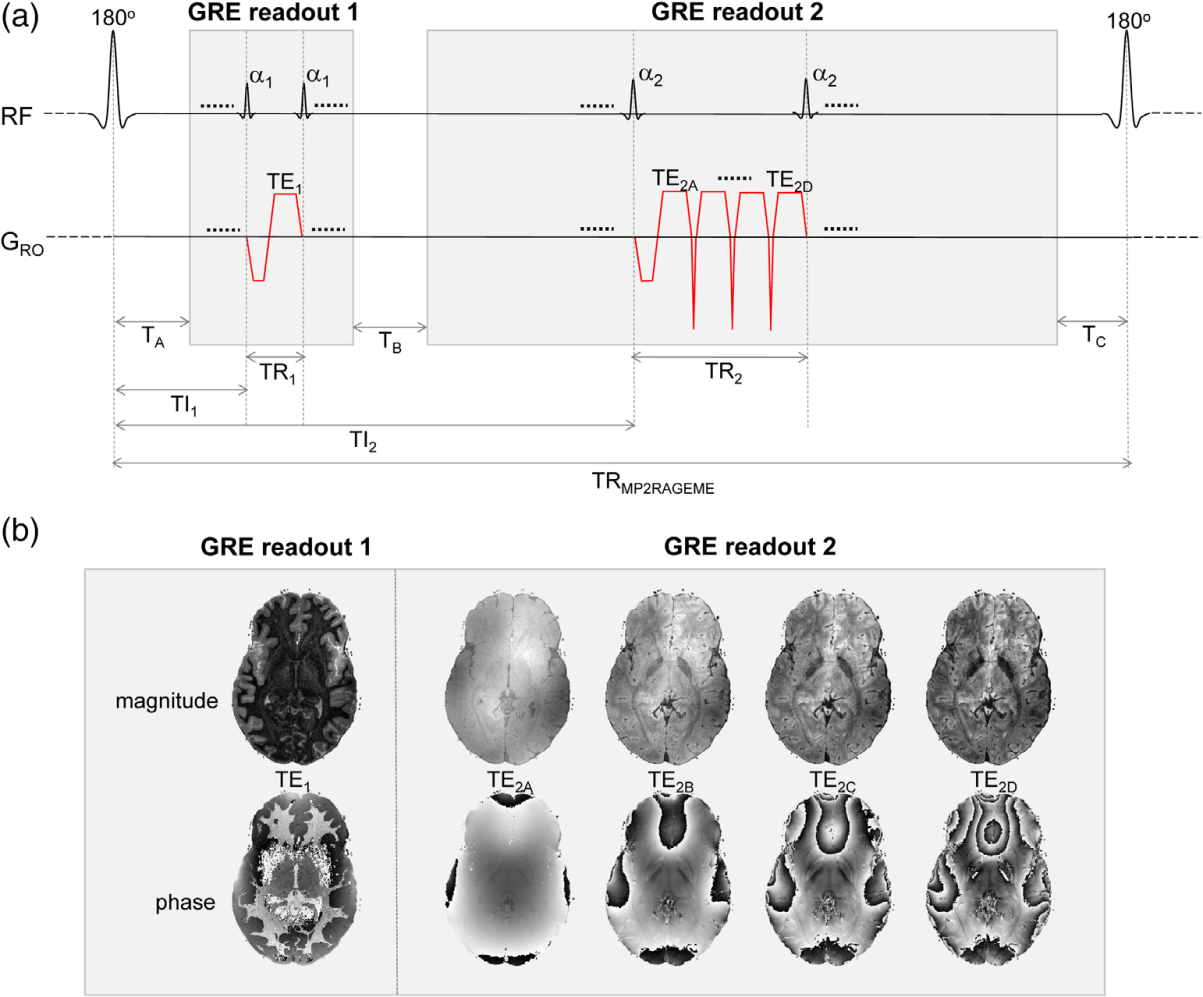
(a) MP2RAGEME sequence, with the second inversion INV_2_ extended to a multi‐echo (ME) readout. The four echoes of INV_2_ have TE's TE_2A‐D_. The TR_GRE_ differs between the first and second inversion images, in contrast with the MP2RAGE sequence. (b) Example magnitude and phase images [Color figure can be viewed at http://wileyonlinelibrary.com]

The MP2RAGE sequence (Marques et al., 2010) was modified so as to acquire ME in the second inversion while maintaining the single‐echo acquisition in the first inversion (Figure 1). Specifically, the longest TE of the second inversion will be designed to be in the range of reported T_2_^*^ values of GM and WM in the brain.

The signal model for the MP2RAGEME sequence for given inversion, echo and repetition times can be analytically written out, as given in the Appendix. T_1_ values were computed from this model through a lookup table with sequence parameter values.

### 2.1 Subjects

Eight healthy volunteers were scanned at 7 T (Philips, Best, NL). The Multiple Interleaved Scanning Sequences environment provided by Philips was used to execute the alternation between the two gradient echo blocks. All subjects provided written informed consent and the study was approved by the local Medical Ethical Committee. From two subjects, the data acquisition was not completed due to hardware failure. For the other six subjects (age range 18–38, four males, and two females), the data were further analyzed.

### 2.2 Sequence

The MP2RAGEME data were compared with separately acquired MP2RAGE and ME‐GRE data in the same session. Parameters in common between all three sequences were: field of view: 205 × 205 × 164 mm, matrix size: 320 × 320 × 256 and hence resolution: 0.64 × 0.64 × 0.64 mm. All imaging slabs were oriented sagittally and rotated 10° around the left–right axis to avoid overlap between the wrapped around signal from the nose and the cerebellum. A SENSE acceleration of 1.7(LR) × 1.7(AP) was used in combination with a circular k‐space window to reduce scan time. The readout bandwidth was 405 Hz for all scans.

We required the longest TE of the second inversion block of the MP2RAGEME to be at least 26 ms, to match the T_2_^*^ of gray matter (Marques & Norris, 2017; van der Zwaag et al., 2016). TE_2A_, the first echo of the second inversion, has to match TE_1_ to be able to cancel out the T_2_^*^ weighting in the T_1_‐weighted MP2RAGE data. A shorter TE results in higher SNR in the T_1_‐weighted image. Hence, TE_2A_ was chosen as short as possible. This led to the range of TE_2A‐D_ of 3/11.5/20/28.5 and a minimal TR_2_ of 31.4 ms. The resulting longer readout of the second GRE block of the MP2RAGEME necessitated that TR_MP2RAGE_ was extended from 6 to 6.7 s. In summary, the following sequences were acquired:

- MP2RAGEME: TR_MP2RAGE_ = 6.72 s, TR_1_ (the repetition time of the first GRE block) = 6.2 ms, TR_2_ = 31.4 ms, TE_1_ = 3 ms, TE_2A‐D_ = 3/11.5/20/28.5 ms, TI_1_/TI_2_ = 670/3855 ms, α_1_/α_2_ = 7/6°. Number of lines/shots per readout: 150, number of readouts: 147. Scan duration: 16:30. Dead time: 6%;
- MP2RAGE: TR_MP2RAGE_ = 6 s, TR = 6.2 ms, TE = 3 ms, TI_1_/TI_2_ = 1,000 ms/3200 ms, α_1_/α_2_ = 7/6°, Number of lines/shots per readout: 150, number of readouts: 147. Scan duration: 14:45. Dead time: 52%;
- ME‐GRE: TR = 31.4 ms, α = 12°, TE_A‐D_ = 3/11.5/20/28.5 ms. Scan duration: 11:35. Dead time: 0%; and
- B_1_^+^‐map: DREAM sequence (Nehrke & Börnert, 2012), voxel size = 8.5 mm^3^, flip angle 60°.

### 2.3 Image processing and quantification

T_2_^*^ maps were obtained from the ME‐GRE and MP2RAGEME data using a single‐exponential fit. QSMs were obtained and averaged from TE_2‐4_ of the ME‐GRE and MP2RAGEME data with respect to cerebrospinal fluid (CSF) using STI Suite (Liu, Li, Tong, Yeom, & Kuzminski, 2015). Here, Laplacian‐based phase unwrapping was performed (Li, Avram, Wu, Xiao, & Liu, 2014). A brain mask was obtained from the first echo TE_1_ magnitude image using SPM8 (Ashburner & Friston, 2005) and eroded by five voxels to remove veins, CSF and regions with low SNR due to susceptibility‐induced intravoxel dephasing. The resulting susceptibility maps were normalized to zero with respect to the whole brain average.

T_1_‐weighted images were corrected for residual transmit B_1_^+^ inhomogeneities using the separately acquired low‐resolution B_1_^+^‐map following (Marques & Gruetter, 2013). Corrected MP2RAGE and MP2RAGEME data were subsequently processed using a dedicated MP2RAGE segmentation pipeline, including skull stripping (Bazin et al., 2014), whole brain segmentation with the multigeometric deformable model (MGDM) algorithm (Bazin et al., 2014; Bogovic, Prince, & Bazin, 2013), cortical reconstruction with the CRUISE algorithm (Han et al., 2004), volume‐preserving cortical depth estimation (Waehnert et al., 2014), and vascular segmentation (Bazin, Plessis, Fan, Villringer, & Gauthier, 2016) to obtain cortical surface reconstructions at three different cortical depths, subcortical and cerebellar regions, and the arterial and venous vasculature around the brain. To explore the cortical parcellations that can be derived from MP2RAGEME‐data, for a single subject, midcortical maps of T_1_, T_2_^*^, and QSM were *z*‐scored parallel to the cortex, allowing for visual inspection of the relative contrast. For quantitative comparison, average distances between the MP2RAGE and MP2RAGEME‐based cortical surfaces over all cortical regions (left and right cerebrum, cerebellum) were computed per subject.

From the MGDM segmentation, median T_1_, T_2_^*^, and QSM values were obtained from the following regions of interest (ROIs): WM (all), nucleus caudate, putamen, thalamus, cortical gray matter (all). For the red nucleus, substantia nigra and subthalamic nucleus (STN), ROIs were defined by coregistration of an atlas, using the maximum probability labels (Keuken et al., 2017), that were thresholded at 10%. The image intensity distributions were subsequently visualized using histograms averaged over all subjects in MATLAB (The MathWorks, Inc., Natick, MA). To assess reproducibility, correlation plots were made and the Pearson correlation coefficient *r* ^2^ was calculated. Bland–Altman plots were generated (Bland & Altman, 1999), and reproducibility coefficients and coefficients of variation were computed, with a Kolmogorov–Smirnov (KS) test on non‐Gaussianity of the difference data.

### 2.4 Simulations

The sensitivity to B_1_^+^ inhomogeneity was simulated by numerically calculating signal intensities using the Bloch equations for both MP2RAGE and MP2RAGEME protocols (Marques et al., 2010). Contrast curves were simulated for B_1_ values of 0.8, 1.0, and 1.2 times the nominal B_1_ value. Also, the amount of T_1_‐induced blurring across the slice direction was simulated for both acquisition protocols. An artificial image was made containing a square of 21 × 21 gray matter voxels (T_1_ = 1.85 s) in a WM (T_1_ = 1.15 s) or CSF (T_1_ = 4 s) background of 150 × 150 voxels, in order to be able to simulate the encoding used in Experiment 1. The signal in each compartment at each excitation during the acquisition of the first and second inversion readouts was computed using Bloch equations. All these images (two inversions times number of phase encoding steps per readout block) were Fourier transformed. Synthetic k‐space data were created where each k‐space encoding line was obtained from the k‐space associated with its actual inversion time. Finally, these were inverse Fourier transformed and combined using the MP2RAGE image combination. The amount of blurring was visualized by comparing the readout direction profile with the slice‐direction profile of the center one‐dimensional image profile.

## 3 RESULTS

For this 0.64 mm resolution protocol, MP2RAGEME offers a 40% time saving over separate scans (16 instead of 26 min) as the ME‐GRE is acquired in the empty time of the TR_MP2RAGE_. The dead time of the MP2RAGE protocol was approximately half the scan time, while that of the MP2RAGEME protocol was only 6%.

**Figure 2:**
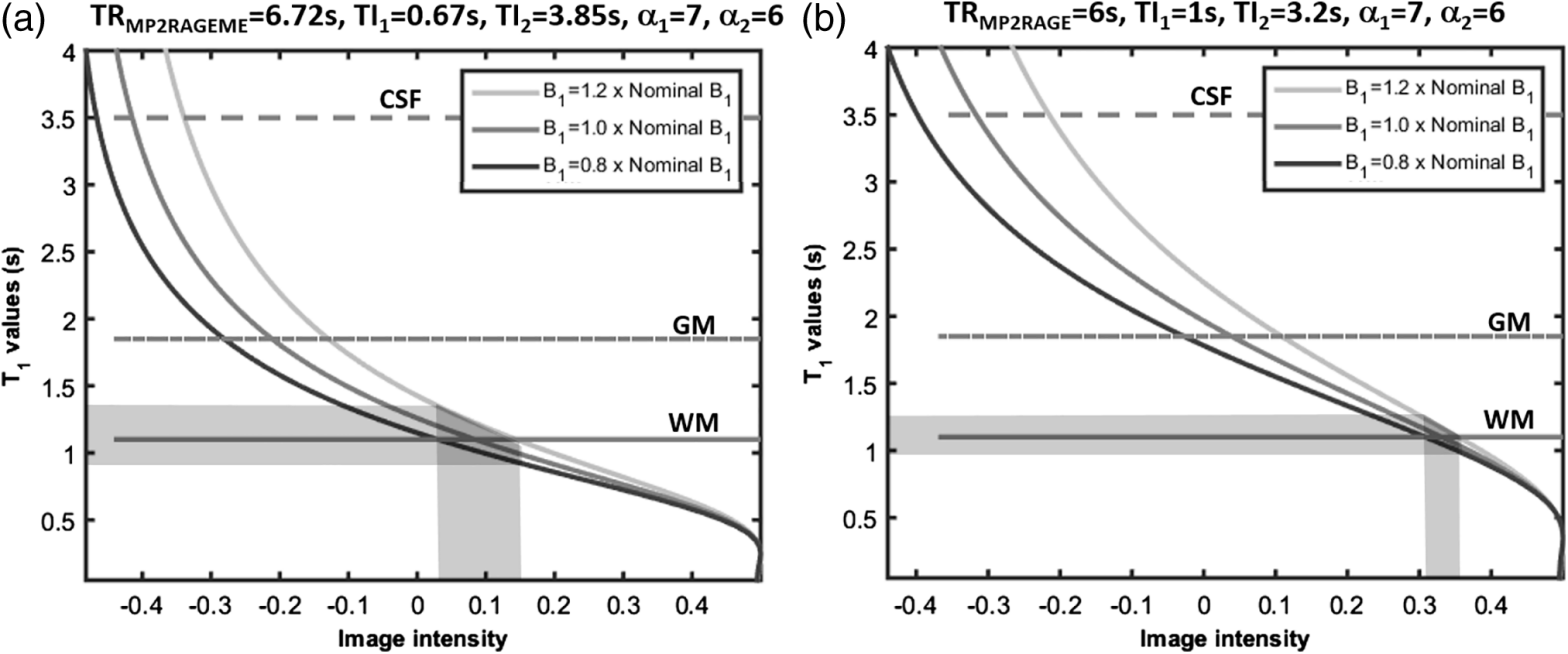
Sensitivity to B_1_^+^‐inhomogeneity. The signal intensity curves of MP2RAGE depend on the local B_1_^+^. The simulated image intensities for the MP2RAGEME (left) and MP2RAGE (right) protocols used are compared here for different B_1_^+^ values. The spread of image intensity values (*x* axis) for the white matter (shaded area, continuous line) is larger in the MP2RAGEME protocol for a B1‐range of 0.8–1.2 times nominal B_1_. For GM (broken line) the image intensity ranges are comparable for the two protocols. For the cerebrospinal fluid (CSF, dotted line), the simulated range of intensities is wider for the MP2RAGE protocol. Note the slightly different ranges of image intensities along the *x* axis

The simulations showed that the B_1_^+^‐sensitivity of the MP2RAGEME‐ and MP2RAGE‐derived T_1_‐weighted images and T_1_‐maps were similar (Figure 2), though there are some differences for the different tissue types. For WM, a wider spread of image intensity values is seen in the MP2RAGEME protocol compared to the MP2RAGE protocol. Comparing 0.8 to 1.2 times the nominal B_1_^+^, the range of intensity values was 0.1 compared to 0.05 AU, and T_1_ ranged from 0.9 to 1.4 versus 1.0 to 1.3, indicating more B_1_^+^‐sensitivity. For GM, the image intensity ranges are comparable and for the CSF the range of intensities is narrower for the MP2RAGEME protocol, indicating equal and less B_1_^+^‐sensitivity, respectively.

**Figure 3:**
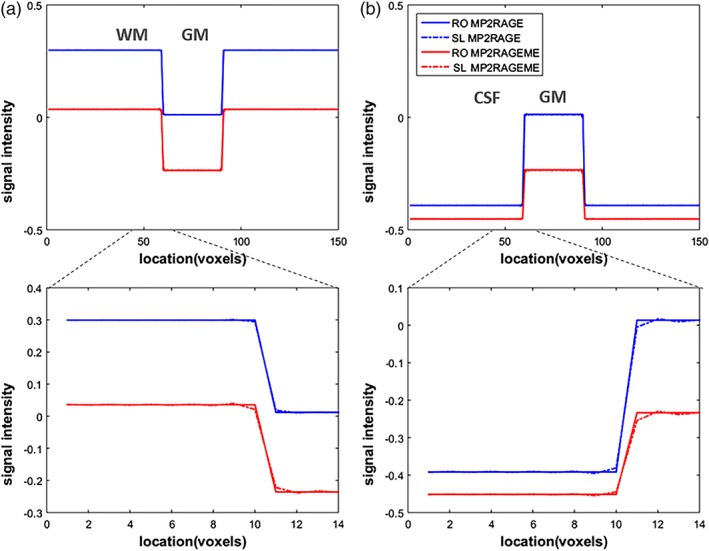
T_1_‐induced blurring. The signal development during the acquisition leads to a blurring in the final image. As most of the T_1_‐evolution happens during the first inversion, this is the main culprit. And as the TI_1_ of the MP2RAGEME is shorter, there is more T_1_‐evolution during the (equally long) readout. This leads to slightly increased blurring in the slice direction (SL, dashed lines), which is compared to the unaffected readout (RO) direction. Note that signal levels differ between the MP2RAGE and MP2RAGEME acquisitions and hence their signal profiles do not overlap. (a) Simulated image containing a square of 30 × 30 GM voxels in a WM background. (b) Simulated image containing a square of 30 × 30 GM voxels in a cerebrospinal fluid (CSF) background. The larger T_1_‐difference between gray matter and CSF means this border is more affected than the WM/GM one [Color figure can be viewed at http://wileyonlinelibrary.com]

The simulations of the amount of T_1_‐induced blurring for both protocols are shown in Figure 3. As the TI_1_ of the MP2RAGEME is shorter than the TI_1_ of the MP2RAGE sequence, here is more T_1_‐evolution expected during the (equally long) readout, and, hence, increased blurring in the slice direction. The simulated slice profiles of the synthetic images show that there is indeed some blurring at the hard boundaries. The blurring is not visible in the complete profiles (Figure 3a,b), but in the zoomed panels in Figure 3b some effects are seen. The MPRAGE protocol has minimal blurring at the WM/gray matter boundary, and shows moderate effects at the larger T_1_‐difference boundary between gray matter and CSF. In the MP2RAGEME differences in signal intensity of the voxels immediately neighboring the boundary can be observed at both frontiers. For both boundaries and acquisition protocols, the contrast‐to‐noise ratio (CNR) necessary to observe such blurring is higher than what is typically achieved in 0.6 mm acquisitions. The CNR for GM/WM was measured to be 10 in one data set, while the blurring induced was less than 10% of the difference between these two tissues.

Regarding the contrast between tissue types, for MP2RAGE‐ME, the GM/WM contrast is maintained. The GM/CSF contrast, that was relatively high in the MP2RAGE sequence, is reduced by 40% to approximately the level of the GM/WM contrast.

**Figure 4:**
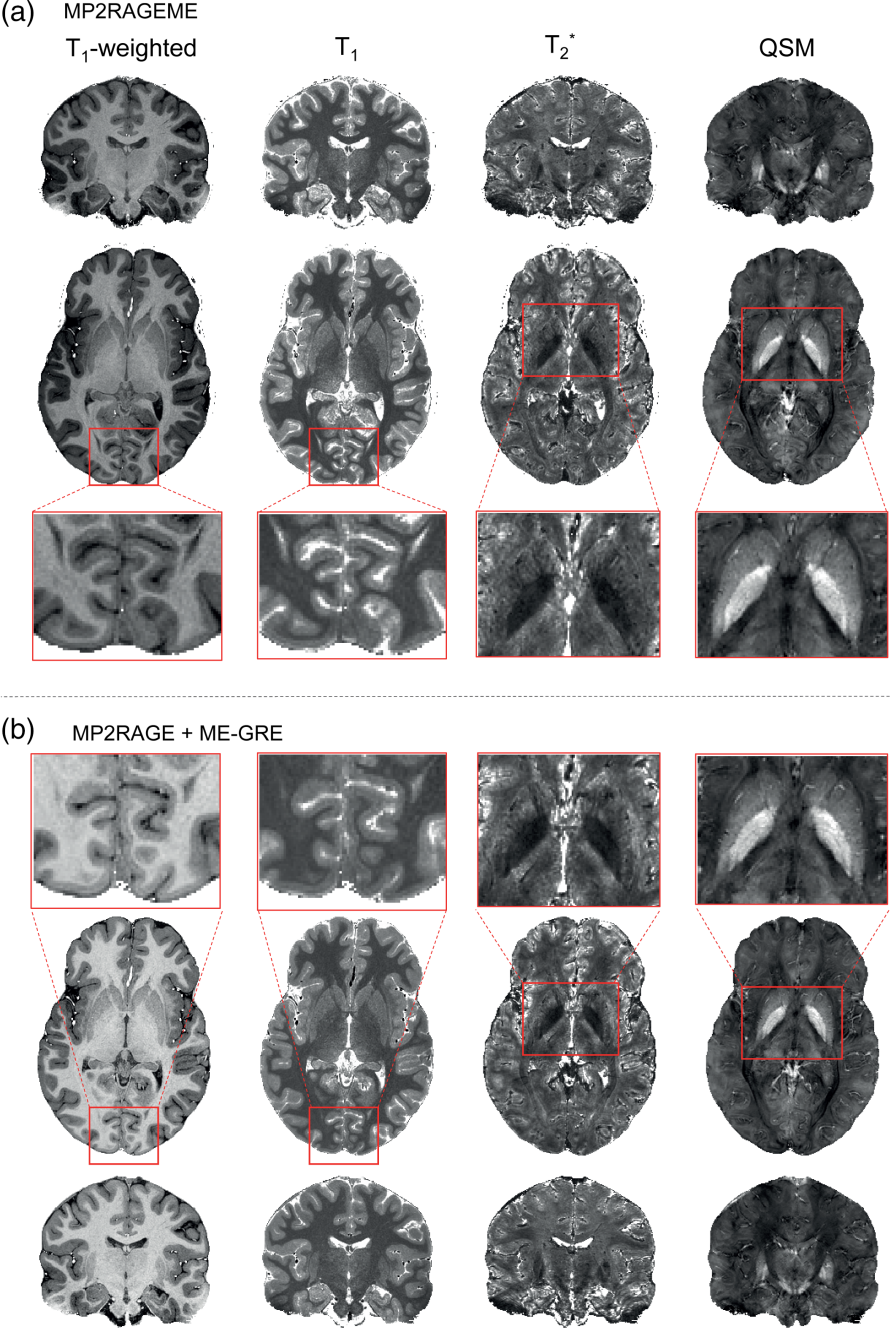
Example derived images. (a) Images generated from the MP2RAGEME acquisition are shown for a single subject (as in Figure 1) in axial and coronal views. T_1_‐weighted images are presented, as are T_1_‐maps, T_2_^*^‐maps, and susceptibility maps. (b) MP2RAGE and ME‐GRE images are shown in coronal view. Note the different T_1_‐weightings but comparable T_1_‐maps [Color figure can be viewed at http://wileyonlinelibrary.com]

**Figure 5:**
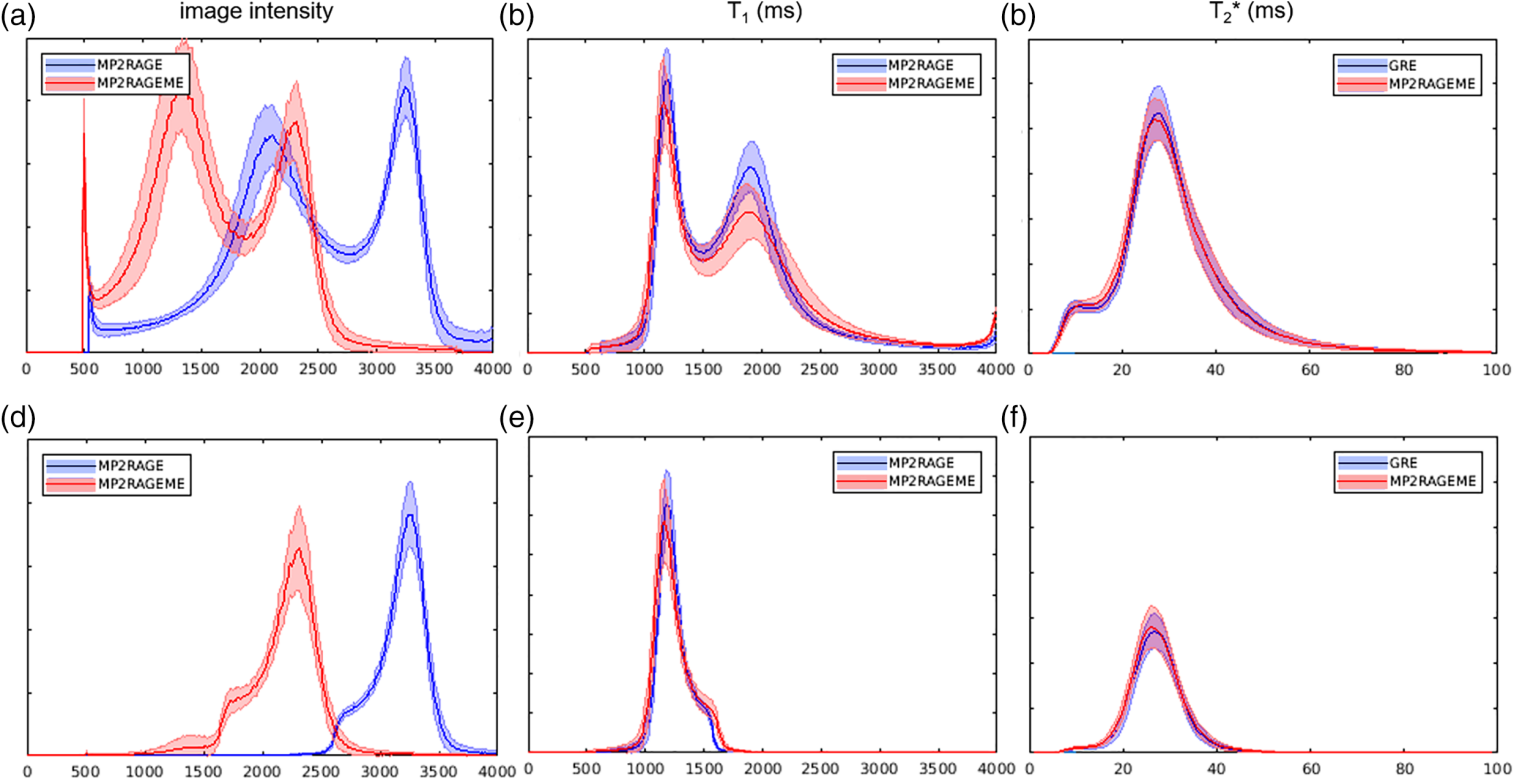
Histograms. The image intensity (a,d), T_1_ (b,e), and T_2_^*^(c,f)‐value distributions of the MP2RAGE, ME‐GRE, and MP2RAGEME acquisitions. Mean values and *SD* (shaded area) over all subjects were computed. Distributions were either taken from the whole brain mask (a–c) or the white matter mask only (d–f). Although the chosen parameters lead to significantly different image intensity distributions (a + d), the derived T_1_‐maps are remarkably similar (b + e). *y*‐Axes represent relative frequencies [Color figure can be viewed at http://wileyonlinelibrary.com]

The image quality overall was excellent for the derived T_1_‐weighted images, as well as for the quantitative T_1_, T_2_^*^, and QSM maps. Figure 4 shows example slices of an MP2RAGEME data set at each orientation for all four derived images, including enlarged sections of specific ROIs in the axial plane. Because of the differences in the acquisition parameters, the T_1_‐weighted image intensity distributions differ significantly between the MP2RAGE and MP2RAGEME. Despite the different signal intensity distributions, the T_1_‐maps showed good agreement (see also Figure 5).

Table 1 summarizes the obtained quantitative T_1_ and T_2_^*^ and QSM values over all volunteers, in comparison with values obtained from the recent literature and from the separately acquired scans (see Figure S1 for an example of the studied ROIs). Most MP2RAGEME‐obtained values were within one *SD* difference to the values obtained from the MP2RAGE and ME‐GRE from the same subjects. For T_2_^*^, the substantia nigra and STN on average differed 1.5 and 1.6 ms, accounting to 10% of the mean reported values. For QSM, the most noticeable effect is the higher variability of 20 ppb in the red nucleus, substantia nigra, and STN. Here, literature values also vary more strongly. Other reported values reside within the range of values reported in literature.

**Table 1:** Mean T_1_, T_2_^*^, and susceptibility (χ) values with *SD* calculated over subjects in ROIs for MP2RAGE‐ME, MP2RAGE, and ME‐GRE sequences, with literature reference values

**T_1_ (s)**	(1)	(2)	(3)	(4)	(5)	(6)	T_1_ (s)	MP2RAGE	MP2RAGEME
WM	1.15 ± 0.06	1.22 ± 0.03	1.13 ± 0.10	1.22 ± 0.03		1.1–1.4	WM	1.22 ± 0.01	1.19 ± 0.02
CN	1.63 ± 0.09	1.75 ± 0.06	1.68 ± 0.07	1.44 ± 0.03	1.68 ± 0.06	1.6–1.7	CN	1.77 ± 0.05	1.80 ± 0.08
Putamen	1.52 ± 0.09	1.70 ± 0.07	1.64 ± 0.16	1.66 ± 0.04		1.5–1.7	Putamen	1.63 ± 0.04	1.63 ± 0.05
Thalamus	1.43 ± 0.10			1.70 ± 0.04			Thalamus	1.59 ± 0.04	1.58 ± 0.04
GM	1.97 ± 0.15/1.87 ± 0.17	2.13 ± 0.10	1.93 ± 0.15	1.80 ± 0.05		1.9–2.1	GM	1.95 ± 0.04	2.00 ± 0.08
RN					1.23 ± 0.04	~WM	RN	1.35 ± 0.08	1.33 ± 0.07
SN					1.31 ± 0.04		SN	1.37 ± 0.09	1.38 ± 0.06
STN					1.19 ± 0.04	~WM	STN	1.30 ± 0.05	1.28 ± 0.04
T_2_^*^ (ms)	(7)	(8)	(9)	(4)	(5)	(6)	T_2_^*^ (ms)	GRE	MP2RAGEME
WM	26.8 ± 1.2			25.9 ± 0.8		24–27	WM	27.1 ± 0.4	26.7 ± 0.1
CN	19.9 ± 2.0			27.4 ± 1.1	28.0 ± 1.9	25	CN	27.5 ± 1.5	27.9 ± 0.5
Putamen	16.1 ± 1.6			33.2 ± 1.9		20–26	Putamen	23.9 ± 1.9	24.1 ± 0.6
Thalamus				30.7 ± 1.9			Thalamus	27.6 ± 1.1	28.1 ± 0.5
GM	33.2 ± 1.3	32.20 ± 1.35	32.2 ± 1.45	23.7 ± 1.8/37.9 ± 2.2		25–33	GM	32.8 ± 1.7	32.4 ± 0.7
RN					17.3 ± 1.5	18	RN	17.3 ± 3.0	16.7 ± 2.4
SN					13.2 ± 1.0		SN	15.6 ± 1.7	14.1 ± 0.9
STN					15.1 ± 1.5	6.0–18	STN	14.8 ± 1.3	16.4 ± 1.4
**χ (ppb)**	(10)	(11)	(12)	(13)	(5)	(6)	χ (ppb)	GRE	MP2RAGEME
WM						−60–20	WM	−8.9 ± 1.1	−6.0 ± 0.7
CN	40 ± 15			44 ± 17		60	CN	32 ± 5	26 ± 2
Putamen	25 ± 20			38 ± 17		20–70	Putamen	29 ± 8	27 ± 1
Thalamus	8 ± 10			45 ± 20			Thalamus	13 ± 5	10 ± 2
GM						0–10	GM	1.3 ± 1.3	1.7 ± 0.2
RN	65 ± 15			100 ± 19	28 ± 36	80–110	RN	74 ± 24	68 ± 22
SN	30 ± 10			152 ± 30	95 ± 34		SN	77 ± 22	75 ± 11
STN				111 ± 27	27 ± 43	80–135	STN	80 ± 22	65 ± 20

CN = caudate nucleus; GM = gray matter; ME‐GRE = multi‐echo gradient echo; RN = red nucleus; ROIs = regions of interest; SN = substantia nigra; STN = subthalamic nucleus; WM = white matter. References: 1: (Marques et al., 2010), 2: (Rooney et al., 2007), 3: (Wright et al., 2008), 4: (Metere et al., 2017), 5: (Keuken et al., 2017), (Marques & Norris, 2017), 7: (Peters et al., 2007), 8: (Cohen‐Adad et al., 2012), 9: (Govindarajan et al., 2015), 10: (Sood et al., 2017), 11: (Langkammer et al., 2012), 12: (Khabipova et al., 2014), 13: (Deistung et al., 2013b).

Figure 5 shows histograms of the image intensities in the MP2RAGEME and separately acquired quantitative maps. Because of the differences in the acquisition parameters, the T_1_‐weighted image intensity distributions differ significantly between the MP2RAGE and MP2RAGEME (Figure 5a,d). In contrast, the T_1_‐maps generated with the appropriate lookup table and following B_1_‐correction are highly similar (Figure 5b,e). Both gray matter and WM peaks are slightly sharper for the MP2RAGE than for the MP2RAGEME. There are negligible differences in the T_2_^*^ values seen (Figure 5c,f).

**Figure 6:**
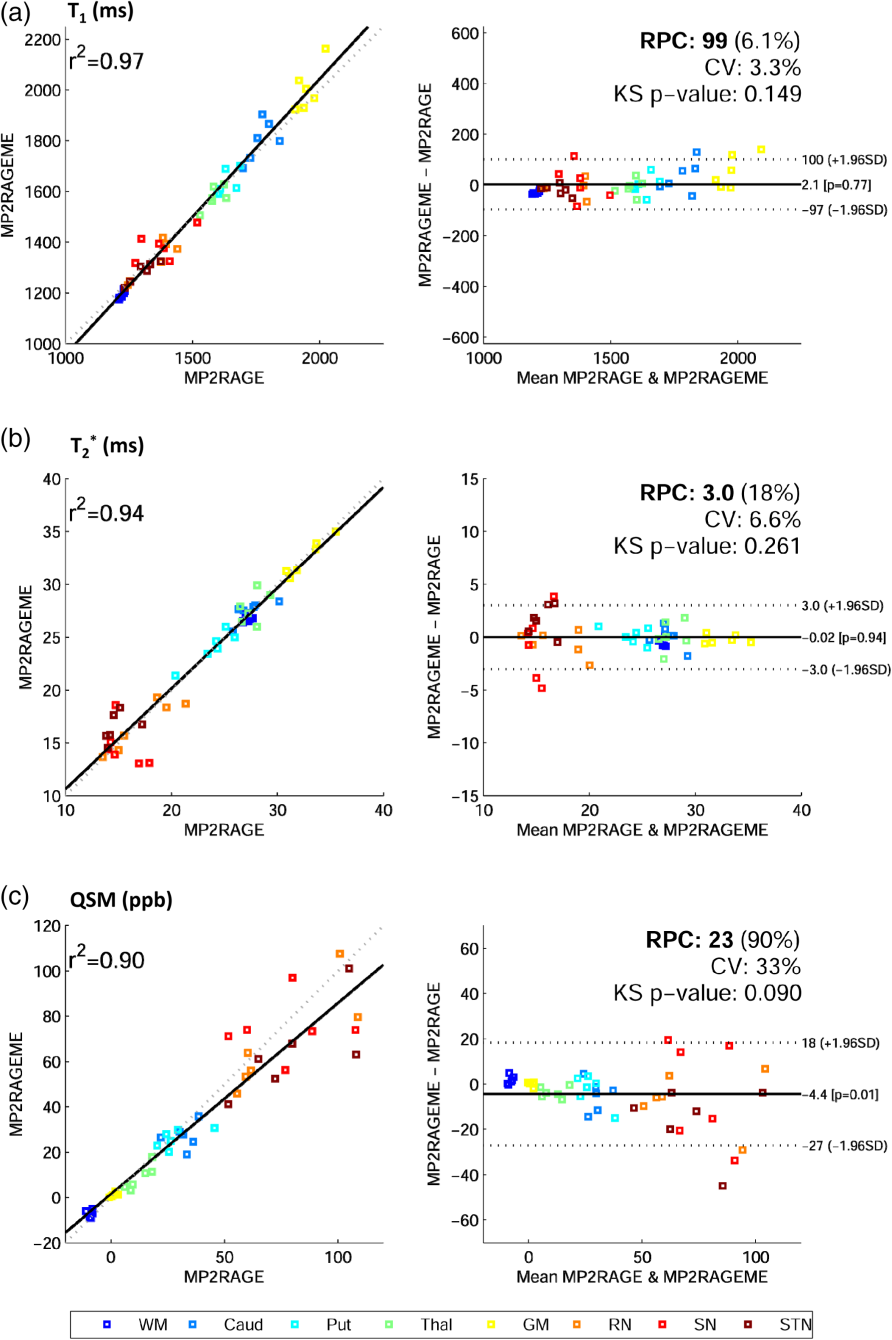
Bland–Altman plots. Correlation and difference plots for (a) T_1_, (b) T_2_*, and (c) QSM, for white matter (WM), caudate (Caud), putamen (put), thalamus (Thal), gray matter (GM), red nucleus (RN), substantia nigra (SN), and subthalamic nucleus (STN). The statistics Pearson's correlation coefficient (*r* ^2^), reproducibility coefficient (RPC), coefficient of variation (CV), and a Kolmogorov–Smirnov (KS) test of non‐Gaussianity of differences are also given. Mean regions of interest values for all six subjects are plotted. Fitted (solid) and identity (dashed) lines are given in the correlation plots (left), and mean difference (solid) and 95% confidence interval (dashed) lines (right) are depicted [Color figure can be viewed at http://wileyonlinelibrary.com]

Bland–Altman plots are displayed in Figure 6, with mean ROI values of all subjects, showing high *r* ^2^ values of 0.97, 0.94, and 0.90 for T_1_, T_2_^*^, and QSM, respectively. Mean differences between sequences were nonsignificant for T_1_ and T_2_^*^ (*p* = .77; *p* = .94) while a small significant difference for QSM was observed (*p* = .01, Δχ =  − 4.4 ppb). No non‐Gaussian data distributions were observed, since KS *p*‐values were .149, .261, and .090 for T_1_, T_2_^*^, and QSM, respectively. Repeatability coefficients reflecting the absolute difference's 95% confidence interval were 99 ms, 3.0 ms, and 23 ppb for T_1_, T_2_^*^, and QSM, the latter being heightened by variable findings in deep brain nuclei, possibly caused by partial voluming or coregistration errors. Also, susceptibility maps are less robust and sensitive to streaking artifacts that can be due to the ill‐posed nature of the problem or originating from phase errors in vessels. The CV was low in T_1_ (3.3%) and T_2_^*^ (6.6%), and higher in QSM (33%), again because of variable results in the deep nuclei. For T_1_, a small positive bias of 50 ms is visible but otherwise similar values were observed, as was the case for T_2_^*^ values throughout the brain. QSM values for caudate, putamen, and thalamus differed by 4 ppb.

**Figure 7:**
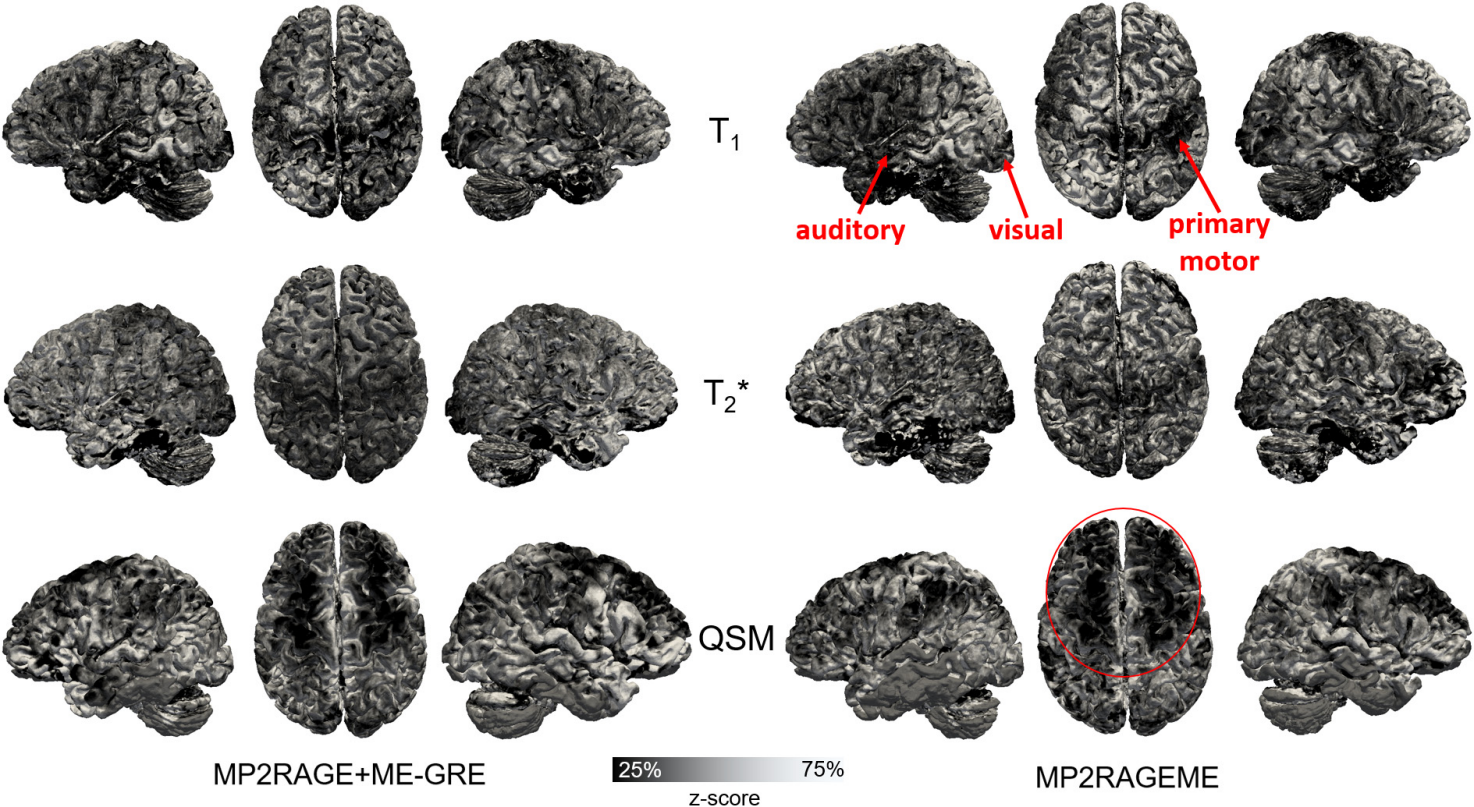
Comparison of the cortical contrasts. Maps of mid‐cortical T_1_, T_2_^*^, and QSM variations obtained for a single subject (as in Figure 1) with the separate MP2RAGE and GREME or the combined MP2RAGEME approach. Cortical depth was estimated with volumetric layering and values were smoothed along the mid‐cortical depth with a 0.64 mm FWHM Gaussian kernel. Surface reconstructions were obtained from the underlying T_1_ maps. To compare local patterns all maps were *z*‐scored parallel to the cortex based on median and interquartile range. Locations of primary motor, auditory and visual cortices are indicated in T_1_ maps, as well as lower QSM values in the anterior part of the cortex [Color figure can be viewed at http://wileyonlinelibrary.com]

Figure 7 shows for a single subject a mapping of T_1_, T_2_^*^, and susceptibility values onto the cortical surfaces. The observed spatial patterns are similar for both sequences, showing, for example, lower T_1_ values in the primary, auditory, and visual motor cortices for both sequences. Furthermore, distinct spatial patterns for the different parameters can be observed, for example, in transversal projections, showing lower T_2_^*^ values in posterior and lower susceptibility values in anterior parts of the cortex, respectively.

The average cortical boundary distances generated from the MP2RAGE and MP2RAGEME data differed over all subjects on average by 0.1 ± 1.1 mm (WM/GM) and 0.1 ± 1.2 mm (GM/CSF). There is thus negligible bias, that is, much smaller than the voxel size and intersubject variation.

**Figure 8:**
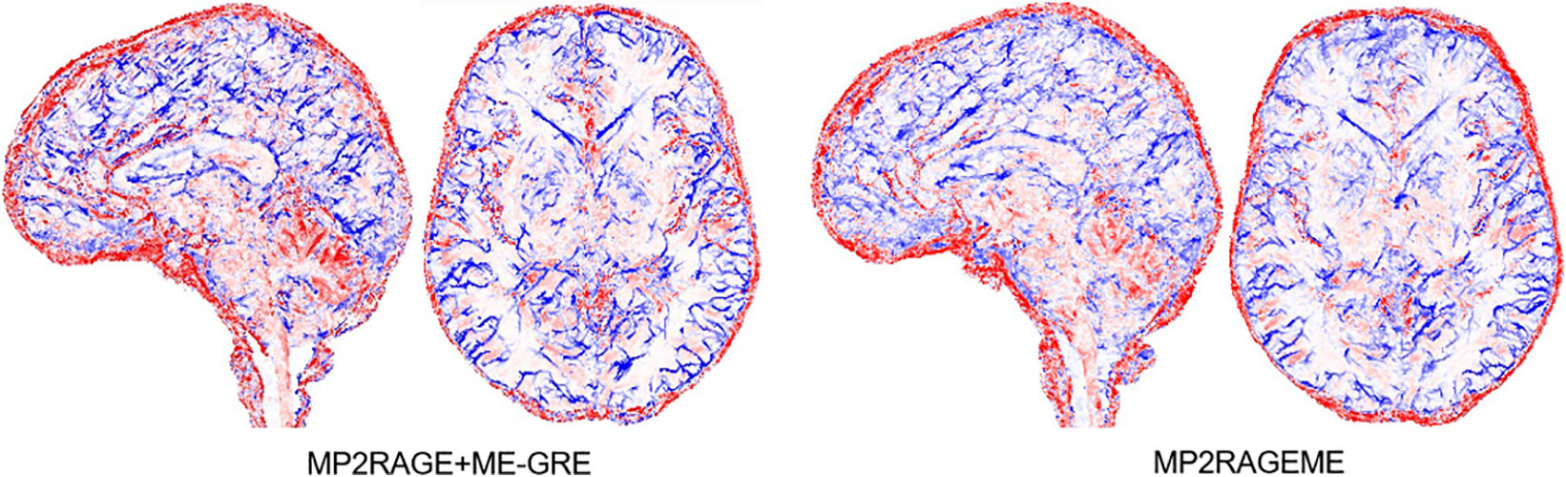
Arterial and venous vasculature. Vasculature reconstructed from the separate MP2RAGE and GREME or the MP2RAGEME‐sequence data (maximum intensity projections over 20 slices in axial and sagittal directions, colored in red for structures extracted from T_1_ maps (arteries) and blue for structures extracted from 1/T_2_^*^ maps (veins). Note the tight interaction of arteries and veins locations, making precise coregistration of the contrast particularly important. Images are for the same single subject as in Figure 1 [Color figure can be viewed at http://wileyonlinelibrary.com]

Figure 8 shows a combined arterial and venous map, by taking the maximum intensity projections of the CBS tools (Cognitive and Brain Sciences tools) software generated vasculature maps. The proximity of the veins and arteries clearly shows the importance of perfect coregistration between the data sets these vessels are derived from. Differences in the vascular trees that are visible between both views are attributed to small motion leading to displacement between subsequently scanned series. Due to this, certain arteries or veins may or may not be included in the ROI used for intensity projection. Also, locally varying shading patterns may be caused by subject motion.

## 4 DISCUSSION

We presented the MP2RAGEME sequence, which allows simultaneous measurement of T_1_, T_2_^*^ and QSM at high resolution. The sequence's two inversion readouts were flexibly designed to have a single echo and ME, respectively, leading to a time‐efficient sequence of 16 min, maintaining a relatively long TE for good T_2_^*^ and QSM contrasts. The reconstructed parameter maps are naturally aligned, allowing for cortical reconstructions and providing parameter values that are in range with reported literature values.

While the optimal parameters for maximum GM/WM and CSF/GM contrast in MP2RAGE can be simulated using the Bloch equations (Marques et al., 2010), the number of parameters to be set is higher in the MP2RAGEME sequence, complicating these simulations. Moreover, as there are multiple contrasts‐of‐interest, the target of such simulations is also not clear (Metere et al., 2017). Hence, we chose sequence parameters for the MP2RAGEME to deliver: (1) optimal contrast in the T_2_^*^ map and QSM data, (2) optimal GM/WM contrast in the first inversion image, and (3) a maximal scan time of 17 min. Further optimization could be achieved by employing a discrete‐time dynamic system to model spin dynamics (Zhao et al., 2018).

Our work builds upon previous work where two identical ME readouts were employed for both inversions (Metere et al., 2017). Here, we could shorten the first readout to a single echo, and lengthen the longest TE of the second readout to 28.5 ms, as compared to 18.91 ms in (Metere et al., 2017), leading to an improved contrast. Our longest TE is thus in line with GRE sequences used elsewhere, reporting 28.4 ms (Deistung et al., 2013b) and 29.6 ms (Forstmann et al., 2014), respectively. Our larger flip angle of the first inversion (7 instead of 4°) resulted in a higher SNR, whereas the higher B_1_^+^‐variation was corrected for by including a B_1_^+^‐map (Nehrke, Versluis, Webb, & Börnert, 2014). Also note that our overall scanning time, although not directly comparable, was slightly shorter, that is, 17 compared to the reported 19 min in Metere et al. (2017), highlighting the time efficiency of the proposed MP2RAGE‐ME sequence.

TI_1_ was chosen to match the TI of a brain‐stem specific protocol (670 ms, (Tourdias, Saranathan, Levesque, Su, & Rutt, 2014)), to facilitate midbrain segmentation using multiple‐contrast data (Bazin et al., 2014). Because the second inversion image cannot overlap in time with the first, the minimum possible value for TI_2_ became nearly 4 ss. The longer TI_2_ reduces somewhat the T_1_‐sensitivity of the T_1_‐weighted images.

The flip angles in the MP2RAGEME of 7 and 6° were set for optimal T_1_ contrast and minimal B_1_^+^‐sensitivity of the T_1_‐weighted images but are lower than the Ernst angle of 12° that was used in the ME‐GRE. This implies that the MP2RAGEME was acquired with a lower SNR than the ME‐GRE, calculated to be 25% lower in WM and 12% lower in GM, respectively. The impact on, for example, manual delineation of deep brain nuclei is thus small.

The shorter TI_1_ resulted in minimally higher B_1_^+^‐sensitivity in the MP2RAGEME T_1_‐map (Figure 2), which was negligible in both MP2RAGE and MP2RAGEME after correction using the DREAM B_1_^+^‐map (Figure 5). Good B_1_^+^‐inhomogeneity correction of both MP2RAGE and MP2RAGEME data is essential for successful cortical segmentation using automated routines (Haast, Ivanov, & Uludağ, 2018).

Both readouts of the MP2RAGE and MP2RAGEME sequences are long relative to the T_1_‐relaxation, meaning that there is significant relaxation during the acquisition of especially the first inversion image. This T_1_‐decay leads to a broadening of the point spread function in the slice encoding direction (Deichmann, Hahn, & Haase, 1999), though this is only a small effect for the protocols compared here (Figure 3).

For the iron‐rich nuclei of the subcortex and cerebellum, the exact colocalization of the three contrasts can help delineate more precise boundaries across the different contrasts, for example, for the globus pallidus or the STN. For the vasculature, a precise colocalization of the arterial (in T_1_ maps) and venous (in T_2_^*^ and QSM maps) vessels was crucial, as these thin and elongated structures often run side by side.

For all ROIs, the T_1_, T_2_^*^, and QSM values derived from the MP2RAGEME or separately acquired MP2RAGE and ME‐GRE are similar. The observed variations are small compared to the spread in literature values. When comparing the quantitative values in Table 1 with the literature values, the only noticeable differences are found for the values reported in the Caudate nucleus, with somewhat longer T_1_ values and lower QSM values found here than in previous work. Other work also noted that the Caudate nucleus and Putamen are difficult to distinguish (Keuken et al., 2014). This might be due to partial inclusion of the CSF in the neighboring ventricles. The relatively large *SD* in QSM values in Table 1, both for the MP2RAGEME and ME‐GRE data, are also observed in the literature references. Still, the susceptibility values obtained from the MP2RAGEME data were similar to those obtained from the separately acquired ME‐GRE data.

Other ROIs were also possibly affected by imperfect segmentation: The red nucleus T_2_^*^ values were shorter than expected from literature (Table 1), possibly due to segmentation errors. Since the WM mask contained subcortical structures, reported QSM values in WM are higher than in the literature. This is also visible in Figure 5f as a small “shoulder” of low‐T_2_^*^ values in the histogram.

The MP2RAGEME sequence is relatively time efficient, with a dead time of only 6%. Since only the second inversion was extended to a ME readout, the first inversion time, which defines the T_1_‐contrast in the MP2RAGE image, can be chosen optimally and total scanning time is shortened compared to an ME readout of both inversions (Metere et al., 2017).

The use of less B_1_^+^ inhomogeneity sensitive radio frequency pulses for the inversion, such as the FOCI pulse, would improve the extent of brain area with homogeneous contrast (O'Brien et al., 2014; O'Brien et al., 2014). The use of SPINS pulses for the echo trains would also yield more homogeneous images, limiting the variation in the ME‐GRE readout due to imperfect flip angles (Malik, Keihaninejad, Hammers, & Hajnal, 2012). Nevertheless, improved B_1_^+^‐homogeneity would benefit these images and, hence, the use of parallel transmission would also be advantageous. On the other hand, if sufficient B_1_^+^ can be achieved throughout the brain, variable flip angle imaging might again show improved performance with minimal point spread function (PSF) blurring.

Subject motion was limited in the experiments performed in this article and did not lead to noticeable image degradation. Only in minimum and maximum intensity projections (Figure 7), locally varying shading patterns can be seen that may be attributed to motion. However, the long, high resolution acquisitions are susceptible to motion artifacts, especially in possible applications to study disease or large populations. Interleaving the acquisition with fat image navigators (Gallichan & Marques, 2017) or using real‐time field control (Özbay, Duerst, Wilm, Pruessmann, & Nanz, 2017) would be candidate approaches to correct for motion artifacts either retrospectively or prospectively.

The proposed algorithm is limited by a longer TR compared to the MP2RAGE sequence, which reduced the CNR between CSF and GM (Figure 3), however, to an acceptable level comparable to the GM/WM contrast. The reduced sequence dead time was accounted for in the signal model, that is, Tc in Equation (1), and thus does not affect T_1_‐quantification. Because of more and longer gradient switching, more heating of the system and consequential resonance frequency drift may occur.

The specific absorption rate (SAR) and a possible raise thereof is of limited concern in MP2RAGE and MP2RAGEME sequences. Because of the low flip angle readout trains, and the inversion pulses being far apart, SAR levels are low for both sequences. If anything the SAR per unit of time is reduced in the MP2RAGEME due to the increased repetition time. This is in strong contrast to, for example, TSE readouts, where much higher flip angles (>100°) are repetitively applied or 2D imaging with simultaneous multi‐slice excitation.

## 5 CONCLUSION

We show that quantitative T_1_, T_2_^*^, and QSM information can be acquired in a single acquisition. Furthermore, we show that the resulting quantitative values are comparable to current separately acquired sequences. Our MP2RAGEME sequence has the benefit in large time savings, perfectly coregistered data and minor image quality differences.

## Supporting information


**Figure S1** ROIs used for analysis, following the color code of Figure 6. Whole brain segmentation with performed the multigeometric deformable model (MGDM) algorithm (Bazin et al., 2014; Bogovic et al., 2013), while cortical reconstruction was done with the CRUISE algorithm (Han et al., 2004).

## Appendix

The signal model for the MP2RAGEME sequence for given inversion, echo and repetition times can be written as:

(1) $$\begin{array}{c} S\left({\mathrm{TI}}_{1},{\mathrm{TE}}_{1}\right)=M_{0}\exp\left(-\frac{{\mathrm{TE}}_{1}}{T_{2}^{*}}\right)\sin\alpha_{1}\\ \cdot \left\{\left[\frac{-\eta M_{z}^{\mathrm{ss}}}{M_{0}}E_{A}+\left(1-E_{A}\right)\right]{\left(E_{1}\cos\alpha_{1}\right)}^{\frac{n}{2}-1}+\left(1-E_{1}\right)\frac{1-{\left(E_{1}\cos\alpha_{1}\right)}^{\frac{n}{2}-1}}{1-E_{1}\cos\alpha_{1}}\right\} \end{array}\;$$

and

(2) $$\begin{array}{c} S\left({\mathrm{TI}}_{2},{\mathrm{TE}}_{2i}\right)=M_{0}\exp\left(-\frac{{\mathrm{TE}}_{2i}}{T_{2}^{*}}\right)\sin\alpha_{2}\\ \cdot \left\{\left[\frac{M_{z}^{\mathrm{ss}}}{M_{0}}E_{C}^{-1}+\left(1-E_{C}^{-1}\right)\right]{\left(E_{2}\cos\alpha_{2}\right)}^{-\frac{n}{2}}+\left(1-E_{2}\right)\frac{1-{\left(E_{2}\cos\alpha_{2}\right)}^{-\frac{n}{2}}}{1-E_{2}\cos\alpha_{2}}\right\} \end{array}$$

with TE_2*i*_ one of the multiple echo times at the second inversion readout only, *M*_0_ the net magnetization, *E*_*j*_ ≡ exp (−TR_*j*_/T_1_) and *E*_*A*_ ≡ exp(−*T*_*A*_/T_1_) and *E*_*C*_ ≡ exp (−*T*_*C*_/T_1_). $\eta \equiv \frac{1}{2}\left[1-M_{z}\left(0^{+}\right)/M_{z}\left(0^{-}\right)\right]$ equals the inversion of the adiabatic inversion pulse, with *M*_*z*_(0^−^) and *M*_*z*_(0^+^) the longitudinal magnetization directly before and after the pulse (Mildner et al., 2014).

$M_{z}^{\mathrm{ss}}$ is the steady‐state longitudinal magnetization (Marques et al., 2010). After introducing a temporary variable *x*

(3) $$x=\left(\left(\left(1-E_{A}\right){\left(E_{1}\cos\alpha_{1}\right)}^{n}+\left(1-E_{1}\right)\frac{1-{\left(E_{1}\cos\alpha_{1}\right)}^{n}}{E_{1}\cos\alpha_{1}}\right)E_{B}+\left(1-E_{B}\right)\right){\left(E_{2}\cos\alpha_{2}\right)}^{n}$$

we can write for $M_{z}^{\mathrm{ss}}$:

(4) $$M_{z}^{\mathrm{ss}}=\frac{M_{0}\left[x+\left(1-E_{2}\right)\frac{1-{\left(E_{2}\cos\alpha_{2}\right)}^{n}}{E_{2}\cos\alpha_{2}}\right]E_{C}+\left(1-E_{C}\right)}{1+\eta {\left(\cos\alpha_{1}\cos\alpha_{2}\right)}^{n}\exp\left(-{\mathrm{TR}}_{\mathrm{MP}2\text{RAGEME}}/T_{1}\right)}$$

The complex‐valued first echo images of both inversion readouts are combined to remove *B*_0_, *M*_0_, and B_1_^+^‐effects, by computing the real part $\mathbb{R}핖\left[\;\right]$ of the normatively averaged signals and *S*^*^ denoting the complex conjugate of *S*:

(5) $$S_{M}=\mathbb{R}핖\left[\frac{S^{*}\left({\mathrm{TI}}_{1},{\mathrm{TE}}_{1}\right)\cdot S\left({\mathrm{TI}}_{2},{\mathrm{TE}}_{21}\right)}{{\left|S\left({\mathrm{TI}}_{1},{\mathrm{TE}}_{1}\right)\right|}^{2}+{\left|S\left({\mathrm{TI}}_{2},{\mathrm{TE}}_{21}\right)\right|}^{2}}\right].$$

T_1_ values were computed from *S*_*M*_ through a lookup table with sequence parameter values (Marques et al., 2010).
