# Supplementary figures and images for: MP2RAGEME: T1, T2 *, and QSM mapping in one sequence at 7 tesla
Source: Hum Brain Mapp. 2018 Dec 13;40(6):1786–98. doi: 10.1002/hbm.24490 (PMC6590660; doi:10.1002/hbm.24490)

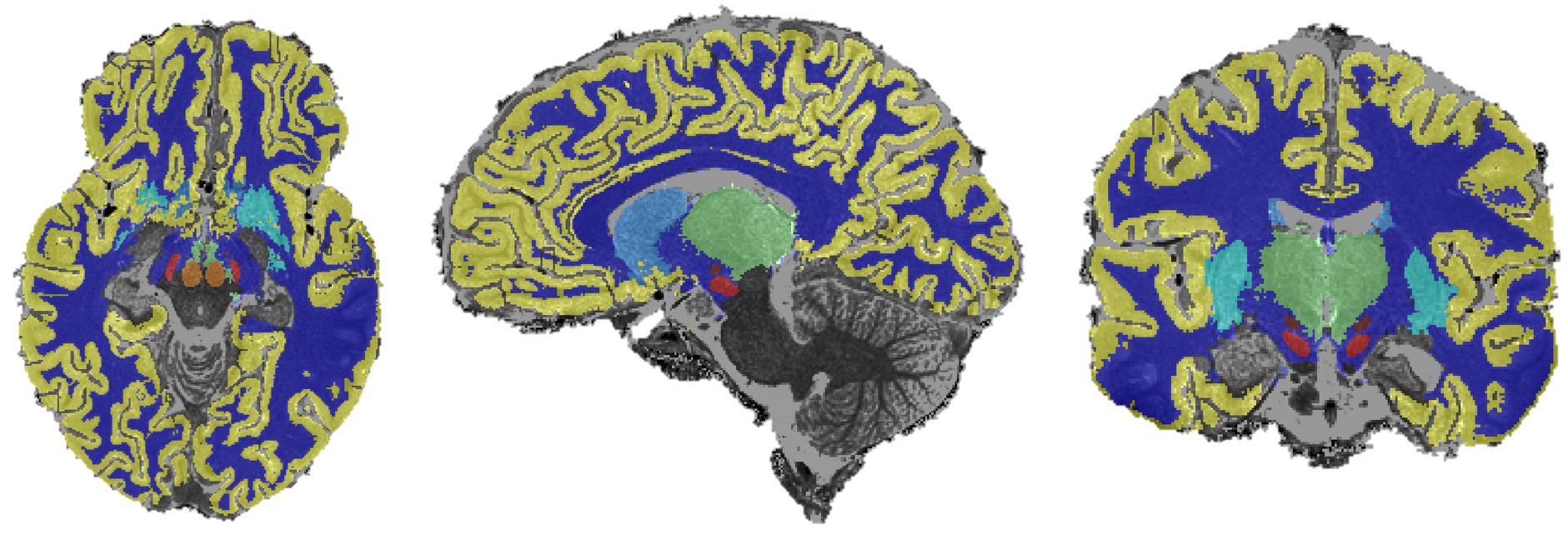

Supplement: Supplementary file 1 — Figure S1 ROIs used for analysis, following the color code of Figure 6. Whole brain segmentation with performed the multigeometric deformable model (MGDM) algorithm (Bazin et al., 2014; Bogovic et al., 2013), while cortical reconstruction was done with the CRUISE algorithm (Han et al., 2004). [file HBM-40-1786-s001.tif]
